# Potential long non-coding RNAs to be considered as biomarkers or therapeutic targets in gastric cancer

**DOI:** 10.3389/fgene.2013.00210

**Published:** 2013-10-18

**Authors:** Mohammadreza Hajjari, Atefeh Khoshnevisan

**Affiliations:** ^1^Department of Genetics, Shahid Chamran University of AhvazAhvaz, Iran; ^2^Department of Genetics, School of Biological Sciences, Tarbiat Modares UniversityTehran, Iran

**Keywords:** long non-coding RNAs, expression level, gastric cancer, oncomine, biomarker

Long non-coding RNAs are attracting the attention of researchers in many fields. These RNAs are longer than 200 nucleotides and do not encode any protein. A subset of them is reported to be strongly correlated with poor patient prognosis, suggesting a potential role in cancer progression (Esteller, 2011).

Gastric cancer is one of the most common malignant tumors worldwide. Therefore, there is an urgent need to find genes which are helpful in the diagnosis, prognosis, and understanding of the molecular pathways of this cancer (Hajjari et al., 2013a).

Finding novel molecular biomarkers of malignancy has been always important for clinical management. Like proteins, mRNAs, and miRNAs, lncRNAs show potentials as novel biomarkers and therapeutic targets in different cancer types (Zhang et al., 2013). However, there are a few reports on studying the relationship between gastric cancer progression and lncRNAs. Some useful clues for further researches seem necessary for deciphering the potential role of lncRNAs in gastric cancer. In this report, we present and highlight some evidences about the potential role of lncRNAs in gastric cancer based on the data indicated in Microarray databases. The results may give novel perspectives for further researches on the role of lncRNAs in gastric carcinogenesis.

We used different databases including Oncomine, Gene Expression Atlas, Gene Expression Omnibus (GEO), and Array Express Databases to analyze the expression level of most recent cited and noted lncRNAs in studies on cancer progression (Niland et al., 2012; Zhang et al., 2013). The selected lncRNAs have been regarded as the most potential biomarkers in the studies on different cancer types (Zhang et al., 2013). The RNAs include *H19, MALAT-1, HOTAIR, ANRIL, CRNDE*, and *MEG3*. Some descriptions of these lncRNAs are presented below.

Oncomine cancer microarray database was used to mine the lncRNA expression profile data according to the established methodology (Rhodes et al., 2007). Four publicly available datasets [Chen Gastric (Chen et al., 2003), DErrico Gastric (D'Errico et al., 2009), Wang Gastric (Wang et al., 2012), and Cho Gastric (Cho et al., 2011)] were inquired for our analysis. By using Oncomine and other microarray expression data accessible from GEO, Gene expression Atlas, and Array Express database, we found some significant differences of expression level for the lncRNAs. The datasets and clinopathological data of each study are presented in Table 1.

**Table 1:** **Different datasets and their clinicopathological data used in the current study**.

**Dataset**	**Clinicopathological data (number of cases)**	**References**
**ONCOMINE DATABASE**		
Chen gastric	Normal tissues (29)	Chen et al., 2003
	Diffuse gastric adenocarcinoma (13)	
	Gastric adenocarcinoma (15)	
	Gastric intestinal type adenocarcinoma (67)	
	Gastric mixed adenocarcinoma (8)	
DErrico gastric	Normal tissues (31)	D'Errico et al., 2009
	Diffuse gastric adenocarcinoma (6)	
	Gastric adenocarcinoma (2)	
	Gastric intestinal type adenocarcinoma (26)	
	Gastric mixed adenocarcinoma (4)	
Wang gastric	Normal tissues(15)	Wang et al., 2012
	Gastric cancer (12)	
Cho gastric	Normal tissues(19)	Cho et al., 2011
	Gastric adenocarcinoma (65)	
	Gastrointestinal stromal tumor (6)	
**GENE ATLAS DATABASE**		
	Normal tissues (31)	D'Errico et al., 2009
	Gastric tumors (38)	
**ARRAY EXPRESS DATABASE**		
E-MTAB–1440	Normal mucosa(20)	Released in Database
	Gastric adenocarcinoma(20)	
**GEO DATABASE**		
GSE22804	Marginal non-malignant tissue (14)	Marimuthu et al., 2011
	Gastric adenocarcinoma (14)	
GSE27342	Normal tissues (80)	Cui et al., 2011
	Gastric cancer (80)	

Student's *t*-test was used for the differential expression analyses. Those which have significant differences (Fold change > 1.5, *P*-value < 0.01) between gastric cancer and normal tissues are brought in Table 2.

**Table 2:** **Significant difference (Fold change > 1.5, *P*-value < 0.01) of the expression level of lncRNAs between gastric cancer types and normal tissues**.

**lncRNA**	**Fold change/T statistics**	***P*-value**	**Category1 (down regulated)**	**Category2 (up-regulated)**
**ONCOMINE DATABASE**				
*HOTAIR*	4.419	3.06E–4	Normal	Diffuse gastric adenocarcinoma
	4.905	1.99E–5	Normal	Gastric intestinal type adenocarcinoma
*H19*	4.396	2.21E–11	Normal	Gastric adenocarcinoma
	2.084	0.003	Normal	Diffuse gastric adenocarcinoma
	3.117	1.16E–6	Normal	Gastric intestinal type adenocarcinoma
*MEG3*	1.572	0.003	Gastric mixed adenocarcinoma	Normal
**GENE ATLAS DATABASE**				
*HOTAIR*	3.2	0.008	Normal	Gastric cancer
*H19*	5.6	5.23E–6	Normal	Gastric cancer
**GEO AND ARRAY EXPRESS DATABASE**				
*H19*	1.5	2.17E–4	Normal	Gastric cancer
	3.84	0.0001	Normal mucosa	Gastric adenocarcinoma
	6.9	0.0001	Marginal non-malignant tissue	Gastric adenocarcinoma

## MALAT-1

We found that the expression level of *MALAT-1* is not significantly different between gastric cancer and normal tissues in the datasets. *MALAT-1* was originally identified to be up-regulated in patients at the high risk of metastasis of non-small cell lung tumors (NSCLC). It seems that *MALAT-1* functions in the regulation of alternate splicing by modulating the activity of SR proteins (Ji et al., 2003).

## ANRIL

There are some reports indicating that *ANRIL* plays an important role in the repression of P14^ARF^, P15^INK4b^, and P16^INK4a^. The proposed mechanism of *ANRIL* is an interaction with Polycomb complexes and silencing target genes. Although some relationships between the *ANRIL* gene and cancer progression have been reported (Yu et al., 2008), we could not find any significant differences of expression between gastric tumor and normal tissues in the datasets.

## CRNDE

*CRNDE* has been identified as an lncRNA whose expression is highly elevated in some tumors. It seems that *CRNDE* is associated with a “stemness” signature in some cancer types (Ellis et al., 2012). For the expression of this lncRNA, we did not find any significant differences between gastric cancer and normal tissues in selected datasets.

## HOTAIR

The datasets show that this lncRNA is up-regulated in gastric adenocarcinoma compared with normal tissues. *HOTAIR* is an lncRNA that binds to the PRC2 complex and targets it at some genes involved in tumor suppression. Its up-regulation is reported in different cancer types (Gupta et al., 2010).

## H19

There is a significant up-regulation in gastric adenocarcinoma tissues compared with normal ones in our results drawn from databases (Table 2). *H19* is an lncRNA which is implicated as having both oncogenic and tumor suppression properties. These two controversial roles of *H19* may be attributed to the nature of *H19* function or context in different tissues. Our results may support its oncogenic role in gastric carcinogenesis.

## MEG3

*MEG3* is an lncRNA which is identified as having an inhibitory role in tumor growth. This lncRNA is the first one proposed to have a tumor suppressor role (Zhang et al., 2003). By querying the databases, we found that the expression level of *MEG3* is down-regulated in gastric adenocarcinoma compared with normal tissues.

Altogether, based on the results, it seems that some lncRNAs such as *HOTAIR*, *H19*, and *MEG3* may potentially be suitable candidates for study in order to find some biomarkers or therapeutic targets. Hajjari et al. found a significant up-regulation of *HOTAIR* lncRNA in gastric adenocarcinoma tissues compared with normal ones by Real-time PCR (Hajjari et al., 2013b). Also, Yang et al. found that *H19* levels were increased in gastric cancer cells and tissues compared with normal controls (Yang et al., 2012). These studies provide supportive evidences concerning the important role of these two lncRNAs in gastric cancer progression.

The different expression of certain cancer type-specific lncRNAs can be exploited for the development of novel biomarkers as lncRNA expression (Gibb et al., 2011). Although there are some reports indicating the effect of down-regulation of *HOTAIR* and *H19* on cell proliferation and metastasis (Yang et al., 2012; Xu et al., 2013), we believe that these functional studies are worth being undertaken on more samples. Besides, understanding their biological roles in normal developments might provide some directions for using these enigmatic molecules as diagnostic or predictive biomarkers.

Our results, drawn from microarray databases, may help researchers to investigate the role of lncRNAs in gastric cancer progression. Large scale and follow-up studies are necessary to consider lncRNAs such as *HOTAIR* and *H19* as potential biomarkers or therapeutic targets in this type of cancer.
